# Complete chloroplast genome sequence of *Zanthoxylum nitidum* (Rutaceae), an important medicinal shrub

**DOI:** 10.1080/23802359.2019.1688725

**Published:** 2019-11-12

**Authors:** Yancai Shi, Rong Zou, Bingbing Liu

**Affiliations:** a Institute of Loess Plateau, Shanxi University, Taiyuan, Shanxi, China;; b Guangxi Institute of Botany, Guangxi Zhuang Autonomous Region and Chinese Academy of Sciences, Guilin, China

**Keywords:** Zanthoxylum, chloroplast genome, phylogenetic analysis

## Abstract

*Zanthoxylum nitidum* (Rutaceae) is a scandent prickly shrub and widely distributed in South China. It’s well known for its valuable medicinal values due to contains some important secondary metabolites. Here, we first report and characterize its complete chloroplast genome based on Illumina paired-end sequencing data. The complete plastid genome was 157,253 bp, which contained inverted repeats (IR) of 27,618 bp separated by a large single-copy (LSC) and a small single copy (SSC) of 84,382 bp and 17,635 bp, respectively. The cpDNA contains 132 genes, comprising 87 protein-coding genes, 37 tRNA genes, 8 rRNA genes. The overall GC content of the plastome is 38.5%. The phylogenetic analysis of 20 selected chloroplast genomes demonstrated that *Z. nitidum* is closely related to the congeneric *Z*. *bungeanum*.

*Zanthoxylum nitidum* (Roxb.) DC., which belongs to the Rutaceae family, is a scandent prickly shrub and widely distributed throughout South China, southeast Asia, and northern Queensland (Kong et al. 1996). It’s a traditional valuable medicine in China. Its stems, leaves and roots have been used as anti-inflammatory and analgesic agents for treatment of toothache, sore throat, stomachache and snakebites (Yang et al. 2008). However, there is very limited information about its genetic and genomic resource. Herein, we first report and characterize its complete plastome based on Illumina paired-end sequencing data, which will contribute to the further studies on its genetic research and resource utilization. The annotated cp genome of *Z. nitidum* has been deposited into GenBank with the accession number MN508801.

In this study, *Z. nitidum* was sampled from in Guangxi Zhuang Autonomous Region of China, located at 108°54′45″ E, 24°57′30″N. A voucher specimen (Y.-C. Shi et al. H146) was deposited in the Guangxi Key Laboratory of Plant Conservation and Restoration Ecology in Karst Terrain, Guangxi Institute of Botany, Guangxi Zhuang Autonomous Region and Chinese Academy of Sciences, Guilin, China. The experiment procedure is as reported in Zhang et al. (2019). Around 2 Gb clean data were used for the cp genome de novo assembly by the program NOVOPlasty (Dierckxsens et al. 2017) and direct-viewing in Geneious R11 (Biomatters Ltd., Auckland, New Zealand). Annotation was performed with the program Plann (Huang and Cronk 2015) and Sequin (http://www.ncbi.nlm.nih.gov/).

The chloroplast genome of *Z. nitidum* is a typical quadripartite structure with a length of 157,253 bp, which contained inverted repeats (IR) of 27,618 bp separated by a large single-copy (LSC) and a small single copy (SSC) of 84,382 bp and 17,635 bp, respectively. The cpDNA contains 132 genes, comprising 87 protein-coding genes, 37 tRNA genes, 8 rRNA genes. Among the annotated genes, 16 of them contain one intron (*atp*F, *ndh*A, *ndh*B, *rps*16, *rpoC*1, *pet*B, *pet*D, *rpl*16, *rpl*2, *rps*12, *trn*A-UGC, *trn*I-GAU, *trn*G-UCC, *trn*K-UUU, *trn*L-UAA and *trn*V-UAC), and two genes (*clp*P and *ycf*3) contain two introns. The overall GC content of the plastome is 38.5%.

To identify the phylogenetic position of *Z. nitidum*, phylogenetic analysis was conducted. A neighbor joining (NJ) tree with 1000 bootstrap replicates was inferred using MEGA version 7 (Kumar et al. 2016) from alignments created by the MAFFT (Katoh and Standley 2013) using plastid genomes of 17 species. It showed the position of *Z. nitidum* was close to the congeneric *Z*. *bungeanum* (Figure 1). Our findings can be further used for plastome evolution and phylogenomic studies of Rutaceae. It will also provide fundamental data for the utilization and management of this important medicinal plant.

**Figure 1.**
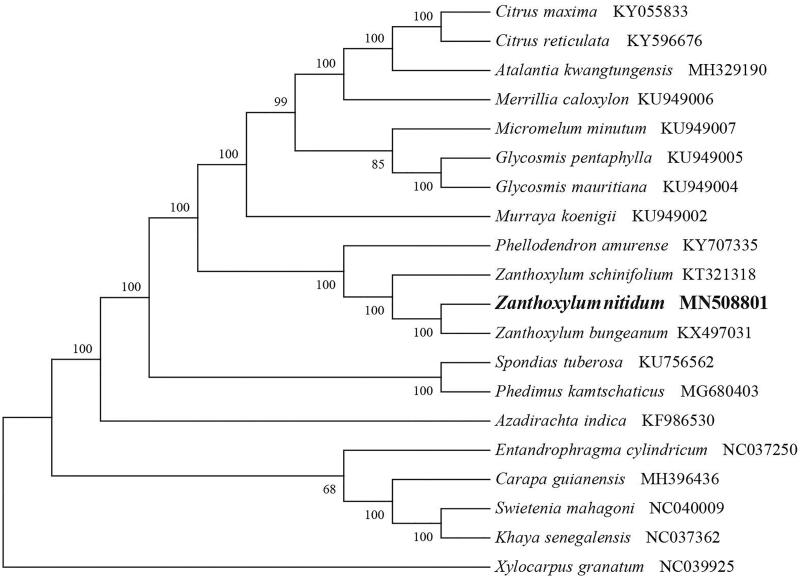
NJ phylogenetic tree of *Z. nitidum* with 19 species was constructed by chloroplast plastome sequences. Numbers on the nodes are bootstrap values from 1000 replicates. *Xylocarpus granatum* was selected as outgroups.
